# National Trends in the Use of Inpatient Hospitalization for Combined Abdominoplasty and Breast Surgery

**Published:** 2015-07

**Authors:** Ashkaun Shaterian, Hossein Masoomi, Jenna B Martin, Keyianoosh Paydar, Garrett A. Wirth

**Affiliations:** Department of Plastic Surgery, Aesthetic and Plastic Surgery Institute, University of California, Irvine

**Keywords:** Abdominoplasty, Breast, Aesthetic surgery

## Abstract

**BACKGROUND:**

Combined procedures involving elective breast surgery at the time of abdominoplasty are frequently performed procedures in aesthetic plastic surgery. While found to be safe outpatient procedures, many surgeons elect to perform combined abdominoplasty/breast surgery as inpatient surgery. This study was performed to explore the practice of performing the combined procedure as an inpatient in the United States.

**METHODS:**

The Nationwide Inpatient Sample database was evaluated using ICD-9CM procedural codes to identify hospitalizations where patients underwent abdominoplasty combined with breast surgery. We trended the frequency of this combined procedure, and evaluated the rate of acute post-operative complications, length of inpatient hospitalization, and total hospital charges.

**RESULTS:**

Between 2004 and 2011, 29,235 combined abdominoplasty/breast procedures were performed as inpatient in United States. The rate of major post-operative complications in the acute hospitalization period was 1.12% and included CVA (0.02%), respiratory failure (0.6%), pneumonia (0.3%), VTE (0.1%), and myocardial infarction (0.1%). Hospitalization averaged 1.8 days and resulted in $31,177 of hospital charges. The demographics of the combined procedure transitioned as i) frequency of inpatient surgeries decreased, ii) percent of patients >50 yr increased, and iii) hospital charges increased from 2004 to 2011.

**CONCLUSION:**

A significant number of surgeons are performing combined abdominoplasty and elective breast surgery as inpatient procedures in United States. The combined surgery is safe but is associated with small risk of major post-operative complications. A short inpatient hospitalization may be beneficial for high-risk patients interested in combined procedures, but must be analyzed against the rising costs of inpatient surgery.

## INTRODUCTION

Abdominoplasty and breast augmentation are frequently requested procedures in aesthetic plastic surgery.^1^ More recently, combined single stage procedures involving breast surgery at the time of abdominoplasty (“Mommy Makeover”) have become a practical option for plastic surgeons.^2^^-^^5^ These combined procedures provide dramatic aesthetic results, single recovery periods, and financial savings, and patients are left more satisfied with their appearance, self-confidence and vitality.^4^^,^^6^ Recent studies have concluded on the safety and utility of these techniques after finding equivalent complications rates for the combined vs. individually performed abdominoplasty/breast procedures.^3^^-^^5^^,^^7^ As such, the combined procedure has become frequently requested by patients and readily offered by plastic surgeons.

Combined abdominoplasty/breast surgery is currently performed in inpatient and outpatient settings.^3^^-^^5^^,^^7^^,^^8^ While some surgeons elect to perform combined procedures as an inpatient surgery with a short hospitalization period, recent studies have found the combined procedure to be safe outpatient surgeries with low rates of complication.^3^^-^^5^^,^^7^^-^^9^ Recent studies estimate that only ~58.2% of all combined abdominoplasty procedures and 65% of all combined breast procedures are performed in office-based suites/ambulatory care centers in the United States.^10^ Older patient age, increased body mass index, lengthy operative time, and extensive blood loss have been cited as risk factors increasing complication rates,^3^^,^^5^^,^^7^^,^^8^ prolonging hospitalization,^11^ and encourage inpatient surgery.

To date, few studies have evaluated the frequency, complication profile, and costs of performing combined abdominoplasty/breast surgery in the inpatient setting. In this study, we used the National Inpatient Sample (NIS) database to evaluate the number of inpatient abdominoplasty/breast procedures being performed in the United States. We evaluated the risk of major complications in the acute post-operative period including myocardial infarction, pneumonia, thromboembolism, respiratory failure, cerebrovascular accidents, and mortality. Lastly, we numerated the length of hospitalization and cost of admission associated with this combined procedure.

## MATERIALS AND METHODS

The NIS is the largest all-payer inpatient care database in the United States. The NIS contains clinical information, resources, and discharge data from over 1,000 hospitals, accounting for nearly 8 million hospital stays each year across the country.^12^ Available from 1988 to 2011 and estimated to encompass as much as 97% of the US population, the NIS has become a clinically useful tool in producing national estimates. Data elements within the NIS allow determination of all procedures performed during a given hospital admission. It also contains discharge information on inpatient hospital stay, including patient characteristics, length of stay, specific in-hospital postoperative complications, total hospital charges and observed in-hospital mortality. 

We conducted a retrospective cross-sectional analysis evaluating the NIS database between 2004 and 2011 using International Classification of Disease 9^th^ revision, Clinical Modification (ICD-9-CM) procedure codes. Our study included patients of all ages, female gender, and those that underwent the combined procedure (abdominoplasty in combination with an elective breast surgery (reduction mammoplasty, mastopexy, and/or augmentation mammoplasty) in an inpatient setting between 2004 and 2011. Patients who had more than one type of breast surgery (i.e. breast augmentation with mastopexy) in addition to an abdominoplasty were also included in our study. 

We numerated the annual frequency of inpatient combined abdominoplasty/breast surgeries between 2004 and 2011. We evaluated the incidence of the combined procedure across patient- and hospital- based factors including age, race/ethnicity, insurance type, geographical location of hospital, type of hospital (teaching vs. non-teaching), costs of hospital stay, and length of hospitalization. Race/ethnicity was categorized as Black, White, Hispanic, Asian, Native American, or other. Insurance type was categorized as Medicare, Medicaid, private, self-pay, no charge, or other. Geographical location within the United States was categorized as West, South, Northeast, and Midwest regions.

Next, we specifically focused on the incidence of major complications occurring in the acute post-operative hospitalization period: (i) Acute respiratory failure; (ii) Venous thromboembolism (VTE); (iii) Myocardial infarction (MI); (iv) Cerebrovascular accident (CVA); (v) Pneumonia; and (vi) Mortality. We evaluated the incidence of complications on an annual basis from 2004 to 2011 and performed a Chi-squared test or Fisher’s Exact test as appropriate. 

Lastly, the length of inpatient hospitalization, total hospital charges, and national distribution of hospital admissions associated with elective inpatient treatment was evaluated. All statistical analyses for the NIS database were conducted using SAS version 9.3 (SAS institute, Cary, North Carolina, USA). Discharge weight (DISCWT) was used to create national estimates for all analysis. Statistical significance was set at *p*<0.05 with all tests two-sided. Approval for this study was obtained from the Human Research Protection (HRP) of the University of California, Irvine Medical Center.

## RESULTS

The NIS database revealed that a total of 29,235 combined abdominoplasty/breast procedures were performed as inpatient surgery between 2004 and 2011 within the United States. Patient characteristics are summarized in Table 1. Patient mean age was 44.0±10.9 years. Overall, 27.7% of patients were older than 50 years of age. 77.5% of patients identified as Caucasian, 9.7% as Hispanic, 7.1% as African American, 1.3% as Asian, 0.5% as Native American, and 4.0% as other. 47.0% of patients had private insurance, 43.2% were self-payers, 4.5% had Medicare insurance, and 1.8% had Medicaid insurance. Geographically, the most common location for combined abdominoplasty and breast surgery procedure was in the Southern United States (36.6%), followed by the Northeast (29.1%), West (22.3%), and Midwest (12.1%). In total, 16,576 (56.7%) combined abdominoplasty/breast procedures were performed at academic teaching hospitals.

**Table 1:** Patient Characteristics

**Age (mean)**	44.0±10.9
Age>50 yr (%)	27.7
**Race **(%)	
White	77.5
Black	7.1
Hispanic	9.7
Asian	1.3
Native American	0.5
Others	4.0
**Payer Type **(%)	
Medicare	4.5
Medicaid	1.8
Private	47.0
Self pay	43.2
Other	3.6
**Geographic Region **(%)	
Northeast	29.1
Midwest	12.1
South	36.6
West	22.3
**Hospital Type **(%)	
Teaching	56.7
Non-teaching	43.3

**Fig. 1:**
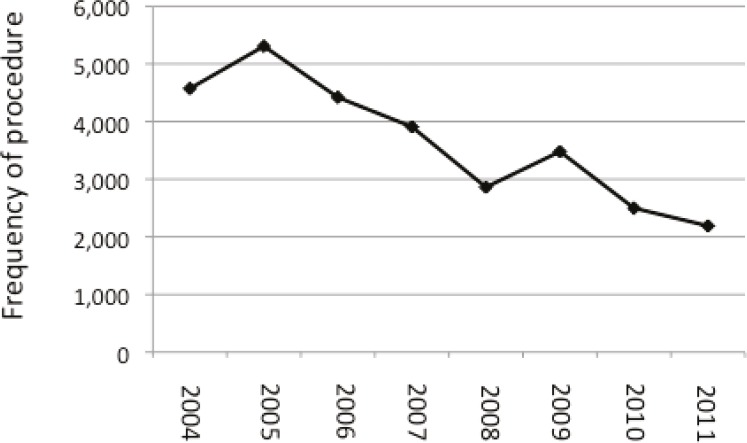
Frequency of inpatient hospitalization following combined abdominoplasty/breast surgery decreased in frequency from 2005 to 2011

To further characterize the use of elective inpatient hospitalization following the combined procedure, we evaluated the annual frequency of elective admissions. We found 29,235 combined abdominoplasty/breast procedures were performed as inpatient, however, the practice of inpatient hospitalization decreased in frequency each year from 2005-2011 (Figure 1). The fewest number of combined abdominoplasty/breast procedures were performed in 2011 comprising 2,190 patients (vs. 5306 performed in 2005) (Table 2).

**Table 2:** Annual trends of combined abdominoplasty and breast surgery in the inpatient setting

	**2004**	**2005**	**2006**	**2007**	**2008**	**2009**	**2010**	**2011**	**Overall**
Combined Procedures	4,572	5,306	4,419	3,908	2,860	3,479	2,498	2,190	29,235

**Fig. 2:**
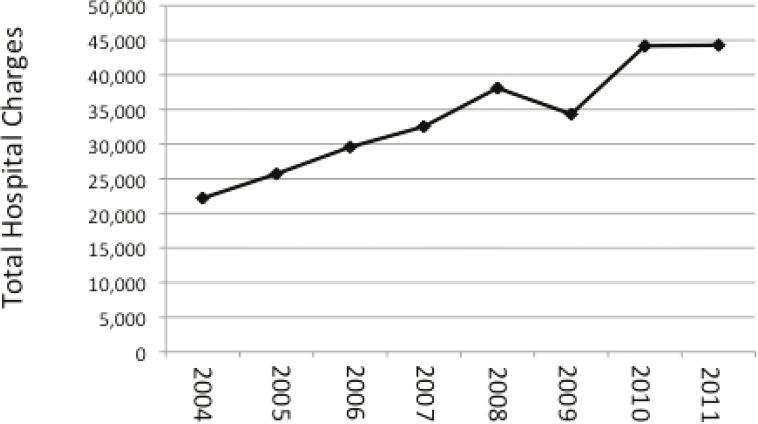
Total hospital charges associated with hospitalization following combined abdominoplasty/breast surgery increased near annually from 2004 to 2011

Length of stay and associated costs of elective inpatient treatment following the combined procedure was evaluated annually from 2004 to 2001 (Table 3). Post-operatively, patients remained admitted for an average of 1.8 hospital days with approximately one of six patients requiring at least 3.2 days of hospitalization. The total hospital charges associated with inpatient admission averaged $31,177 for 1.8 day admission; however the top 15% of admissions reached above $56,108. Interestingly, while mean hospital stay demonstrated minimal variation from 2004-2011, hospital charges increased near annually from 2004 ($22,194) to 2011 ($44,302) (Figure 2). 

**Table 3:** Length of hospital stay (LOS) and total hospital charges (THC)

	**2004**	**2005**	**2006**	**2007**	**2008**	**2009**	**2010**	**2011**	**Overall**
LOS (days)	1.8	1.7	1.8	1.9	1.9	1.8	1.9	1.8	1.8±1.5
THC ($)	22,194	25,706	29,578	32,536	38,099	34,332	44,186	44,302	31,177±24,931

The incidence of acute post-operative complication of combined abdominoplasty/breast procedures that may warrant inpatient hospitalization was evaluated revealing a 1.12% overall major complication rate (Table 4). The most frequent major complications included pneumonia (0.3%) and acute respiratory (0.6%) failure. However, we also found 1 of 1000 patients suffered a VTE, 1 of 1000 suffered a MI, and 1 of 5000 suffered a CVA within the acute post-operative period. The mortality rate was 0.0% in the acute period following the combined procedure. We found the rate of complication varied by year (*p*<0.05) but did not follow an annual trend. Taken together, this data suggests that combined abdominoplasty/breast procedure is an overall safe procedure, but does present a small risk of serious post-operative complications.

**Table 4:** Incidence of major post-operative complications following combined abdominoplasty/breast surgery

**Complication**	**2004**	**2005**	**2006**	**2007**	**2008**	**2009**	**2010**	**2011**	**Overall**	**P value**
Pneumonia	0.4	0.0	0.3	0.4	0.5	0.2	0.6	0.4	0.3	<0.01
ARF	0.3	0.5	0.6	0.9	0.5	0.9	0.2	0.6	0.6	<0.01
VTE	0.0	0.1	0.0	0.4	0.2	0.1	0.2	0.2	0.1	<0.01
MI	0.0	0.1	0.0	0.0	0.0	0.0	0.2	0.2	0.1	<0.01
CVA	0.0	0.0	0.0	0.1	0.0	0.0	0.0	0.0	0.02	<0.01
Mortality	0.0	0.0	0.0	0.1	0.0	0.0	0.0	0.0	0.0	0.02
Total	1.12									

* ARF: Acute respiratory failure, VTE: Venousthromboembolism, MI: Myocardial Infarction, CVA: Cerebrovascular Accident

## DISCUSSION

In the present study, we evaluated national data using the NIS database and found 29,235 combined abdominoplasty/breast (Mommy Makeover) procedures were performed on an inpatient basis in the United States between 2004 and 2011. This combined abdominoplasty/breast procedure resulted in a 1.12% rate of serious complication in the acute post-operative period. Patients were admitted for an average of 1.8 days, resulting in an average of $31,177 in hospital charges. We found the demographics of the combined abdominoplasty/breast procedure captured in the NIS database transitioned as the frequency of inpatient surgeries decreased, percent of patients  >50 years old increased, and total hospital charges increased from 2004 to 2011.

Currently, little is known about the use of elective inpatient hospitalization following combined abdominoplasty/breast surgeries. While numerous surgeons perform these combined procedures on an outpatient basis,^3^^-^^5^^,^^7^^,^^8^ other providers elect to admit their patients post-operatively^11^ (Table 2). The data presented in this study reveals that a significant number of combined procedures are being performed in an inpatient setting. The TOPS (Tracking Operations and Outcomes by Plastic Surgeons) database has revealed that only ~58.2% of all combined abdominoplasty procedures and 65% of all combined breast procedures are being performed in office-based suites/ambulatory care centers in the United States.^10^ Studies have found patient- and surgery-specific variables (patient age, body mass index, operative time, and estimated blood loss) to represent risk factors increasing complication rates,^3^^,^^5^^,^^7^^,^^8^ prolonging hospitalization,^10^ and encourage inpatient surgery. The decision for elective admission may also reflect provider concerns over post-operative pain control, mobility assistance, or risk of a serious complication for higher risks patients. Despite the frequency of elective admission post operatively, we found this practice decreased from 2004 to 2011, which may reflect recent publication promoting the safety of combined procedures in outpatient settings.^3^^-^^5^^,^^7^^,^^8^

Patient and surgery specific variables are well-established factors affecting the decision to perform elective surgery. In this study, we found the proportion of patients older than 50 years undergoing inpatient abdominoplasty/breast surgery increased yearly from 2004 to 2011. This may represent an older population interested in or healthy enough to undergo the combined procedure due to surgical advances. However it may reflect surgeons’ decision to admit more at-risk patients. It was shown that age to be a significant factor after finding 60.5% of patients >40 yr (vs. 37.1% of patients <40 yr) necessitated prolonged hospitalization and additional follow up visits following combined procedures.^10^ Elderly patients are a higher risk population with additional comorbidities and may benefit from less sophisticated techniques, limited abdominoplasty resections in combined surgery, and elective inpatient admission.^11^^,^^12^

The safety of combined procedures and incidence of post-operative complications have been of interest since the introduction of single stage abdominoplasty/breast surgery. In this study, we evaluated over 29,000 combined abdominoplasty/breast procedures and found the incidence of major complications in the acute post-operative period to be 1.12%. Similar to previously published studies,^3^^,^^5^^,^^8^ we found a low rate of VTE (0.1%) and mortality (0.0%). However, this study is one of the few to evaluate the incidence of CVA (0.02%), acute respiratory failure (0.6%), and myocardial infarction (0.1%) following the combined procedure. Ultimately, combined abdominoplasty/breast procedure is associated with a low risk of major complications and may require inpatient surgery for higher risk patients.

Numerous studies have commented on the safety of the combined procedure as an outpatient surgery. Inpatient surgery provides patients with acute care services, but often with higher associated facility charges. We found the average hospital charge to be $31,177 for an average 1.8 day hospital stay; these costs increased near annually from 2004 to 2011 until reaching $44,302. We hypothesize that this may reflect increasing surgeon, anesthesia, or hospital fees, advancing technology, pre-operative work up, or the possibility of a reporting system issue that does not account for negotiated cosmetic fees. The advancing age of patients undergoing the procedure or selective admission for high-risk patients may also be increasing costs. Considering the risk of major complications in the acute post-operative period, inpatient surgery may serve as a safe alternative for patients at higher risk due to age, presence of comorbidities, or lack of outpatient support. Ultimately, the benefits of inpatient admission must be analyzed against the increasing costs of acute post-operative care.

There are several limitations to this study. First, our study aimed to evaluate serious complications occurring in the acute inpatient setting. Therefore, our study does not represent a complete evaluation of minor post-operative complications (i.e. hematoma, infection, etc). We also did not categorize the type of abdominoplasty, type of elective breast surgery procedure (reduction, augmentation, or mastopexy), surgical technique, or operating time which may have affected complication rates, length of hospital stay, and hospital charges. The NIS database identifies important demographic data from the discharge abstract, but does not include information on post operative management including VTE prophylaxis, pain control regimens, or antibiotics. Despite these limitations, this study identified important trends on a national level in the practice of inpatient abdominoplasty/breast surgery.

Combined procedures are becoming a frequently employed surgical option for patients across various surgical fields. While providing numerous benefits to patients due to its single stage nature, combined surgeries have the potential for added risks and complications. To date, few studies have evaluated patient- and surgery- specific risk factors that increase post-operative complications that may necessitate inpatient admission. To this end, continued research is needed in the area of combined surgeries to ensure patient safety and optimal surgical outcomes. 

Using the NIS database, we found the use of inpatient surgery for combined abdominoplasty/breast surgery is decreasing in the United States but is still being performed in significant numbers. The combined procedure appears to be a safe surgical option but is still associated with a risk of major complications (MI, VTE, CVA, ARF) in the acute post-operative period. Inpatient surgery may serve as a safe alternative for higher risk patients, but must be analyzed with the rising costs of inpatient admission.

## CONFLICT OF INTEREST

The authors declare no conflict of interest.

## Note

Please cite this paper as:

Shaterian A, Masoomi H, Martin JB, Paydar K, Wirth GA. National Trends in the Use of Inpatient Hospitalization for Combined Abdominoplasty and Breast Surgery. World J Plast Surg 2015;4(2):120-126.
